# Rabies in the Baltic States: Decoding a Process of Control and Elimination

**DOI:** 10.1371/journal.pntd.0004432

**Published:** 2016-02-05

**Authors:** Emmanuelle Robardet, Evelyne Picard-Meyer, Marianna Dobroštana, Ingrida Jaceviciene, Katrin Mähar, Zita Muižniece, Gediminas Pridotkas, Marius Masiulis, Enel Niin, Edvīns Olševskis, Florence Cliquet

**Affiliations:** 1 ANSES, Nancy Laboratory for Rabies and Wildlife, Bâtiment H, Technopôle Agricole et Vétérinaire, CS 40 009, Malzéville, France; 2 BIOR, Institute of Food Safety, Animal Health and Environment "BIOR", Riga, Latvia; 3 National Food and Veterinary Risk Assessment Institute of Lithuania, Vilnius, Lithuania; 4 Vilniaus Kolegija/University of Applied Sciences Faculty of Agrotechnologies, Buivydiškės, Vilnius district, Lithuania; 5 Estonian Veterinary and Food Laboratory, Tartu, Estonia; 6 State Food and Veterinary Service, Vilnius, Lithuania; 7 Estonian Veterinary and Food Board, Tallin, Estonia; 8 Food and Veterinary Service, Riga, Latvia; Swiss Tropical and Public Health Institute, SWITZERLAND

## Abstract

Rabies is a fatal zoonosis that still causes nearly 70, 000 human deaths every year. In Europe, the oral rabies vaccination (ORV) of red foxes (*Vulpes vulpes*) was developed in the late 1970s and has demonstrated its effectiveness in the eradication of the disease in Western and some Central European countries. Following the accession of the three Baltic countries—Estonia, Latvia and Lithuania—to the European Union in 2004, subsequent financial support has allowed the implementation of regular ORV campaigns since 2005–2006. This paper reviews ten years of surveillance efforts and ORV campaigns in these countries resulting in the near eradication of the disease. The various factors that may have influenced the results of vaccination monitoring were assessed using generalized linear models (GLMs) on bait uptake and on herd immunity. As shown in previous studies, juveniles had lower bait uptake level than adults. For the first time, raccoon dogs (*Nyctereutes procyonoides)* were shown to have significantly lower bait uptake proportion compared with red foxes. This result suggests potentially altered ORV effectiveness in this invasive species compared to the red foxes. An extensive phylogenetic analysis demonstrated that the North-East European (NEE) rabies phylogroup is endemic in all three Baltic countries. Although successive oral vaccination campaigns have substantially reduced the number of detected rabies cases, sporadic detection of the C lineage (European part of Russian phylogroup) underlines the risk of reintroduction via westward spread from bordering countries. Vaccine induced cases were also reported for the first time in non-target species (*Martes martes* and *Meles meles*).

## Author Summary

This paper reviews ten years of rabies epidemiology in the three Baltic countries. Both surveillance efforts and oral rabies vaccination campaigns have resulted in the near eradication of the disease. Multivariate analysis assessed with generalized linear models (GLM) suggested lower oral vaccination effectiveness in raccoon dogs compared with red foxes, highlighting the importance of adapting oral vaccination strategy to each vector of the disease. Although eradication of the disease is almost achieved, the detection of some cases belonging with the Russian rabies lineage emphasizes a risk of rabies reintroduction in the Baltic States due to westward spread from bordering countries. This study show also the first vaccine-induced cases detected in non-target species (*Martes martes* and *Meles meles*).

## Introduction

Rabies disease is a fatal mammalian encephalomyelitis caused by the rabies virus of the genus Lyssavirus (family Rhabdoviridae) [1]. The virus is distributed worldwide, with the exception of the Antarctic, Australia and several islands and although all species of mammals are susceptible to this virus, it infects principally carnivores and bats [2]. In Europe, the genus lyssavirus evolves through five virus species (four of them circulate in bats only): the classic rabies virus (RABV) affecting non-flying terrestrial mammals only, the european bat lyssaviruses type 1 and type 2 (EBLV-1 and EBLV-2) and the more recently detected Bokelo bat lyssavirus (BBLV) and Lleida bat lyssavirus not yet taxonomically assessed [3].

RABV has spread in Europe since antiquity as a dog and wolf-mediated disease [4]. In the 1940s, likely due to spillover from domestic animals, a new epizootic maintained by a single species, the red fox, emerged in Eastern Europe with an assumed ground zero in Kaliningrad [5].The front moved from Poland to Germany spreading through Europe with a speed of approximately 30–60 km per year, reaching France in 1968 and Italy in 1980 [6]. Large rivers, lakes and high mountain chains acted as obstacles to the spread; bridges facilitated the crossing of rivers. Intensive fox destruction campaigns alone cannot stop the spread of the virus [7], prompting oral rabies vaccination (ORV) programs that rapidly proved to be the only efficient technique for controlling the disease. The first ORV field trial was conducted in 1978 in Switzerland [8] and was gradually extended to surrounding countries, such as Belgium, France and Germany. In the 1980s, fox rabies control in European Union became a public health issue. Since 1989, the European Commission has provided funding to Member States for national eradication programmes, thereby improving surveillance and encouraging regular implementation of oral vaccination campaigns on large scales in coordination with neighbouring countries. This strategy leaded to the successful elimination of rabies in most Western and Central European countries [9,10]. In Europe, approximately half of the historical rabies endemic countries are now free of rabies (Austria, Belgium, Czech Republic, Finland, France, Germany, Italy, Luxembourg, Switzerland and the Netherlands). In the Baltics, the three countries were recently officially recognized free of rabies according to OIE (World Organisation for Animal Health) criteria [11–13]. In the last three years, some sporadic cases have been reported in some countries (Bulgaria, Hungary, Slovakia and Slovenia) and the disease is still endemic in several Eastern European countries (eastern Poland, Romania, Ukraine, Belarus and Russia, source: http://www.who-rabies-bulletin.org/Queries/Surveillance.aspx).

In the Baltic States, represented by Lithuania, Estonia and Latvia, sylvatic rabies emerged in the 1950s-1960s [14]. Since this time, a surveillance of the disease were progressively implemented and positive cases have been observed mainly in red foxes and raccoon dogs [15–17]. Although the red fox is known to be highly susceptible to RABV and is the main reservoir and vector of rabies throughout Europe, the Baltic countries has the particularity to host a second vector and reservoir, i.e. the raccoon dog [14]. Raccoon dog is one of the most successful alien carnivores in Europe. Native to East of Asia, this species was introduced in the eastern part of Russia via fur industry during the first half of the 20^th^ century and has spread throughout Europe, becoming common in the Baltics and some other northeastern European countries. After it was first observed in the 1950’s in the Baltics, ten years were required to colonize the entire countries [18]. Foxes and raccoon dogs are both opportunistic omnivores, often share the same habitats and overlap their home ranges increasing the probability of contacts between the two species. Moreover, their combined densities could allow rabies epizootics to persist in a certain area [19]. The existence of this important rabies transmitter in this area challenged health authorities and questioned on its potential impact on the success of conventional ORV method used to control rabies in Western Europe.

ORV programs were experimented differently according to the Baltic State. While no ORV was implemented in Estonia until 2005, in Latvia, ORV was firstly initiated in 1991 using chicken head vaccine baits. ORV using manufactured baits started in 1998 and has been performed twice a year since 1999, but regular purchase of the necessary amount of vaccine baits for annual nationwide vaccination was not possible because of financial reasons. The vaccination area was enlarged every year to cover the whole territory by 2001–2003 and vaccines were distributed manually [20]. In Lithuania, ORV was tested for the first time in 1983 with fish or meat baits containing a vaccine made of a derived ERA (Evelyn Rokitnicki Abelseth) laboratory fixed virus strain produced in Russia. In 1993 ORV was occasionally assessed on three districts [21]. Between 1995 and 2000, following the Lithuanian National Rabies prevention programme, ORV was performed generally manually and a large range of vaccines was used (Street Alabama Duffering (SAD) Bern, SAD P5/88 (Rabifox), (Street Alabama Gif (SAG) 1) over variable geographic areas.

Following the accession of three Baltic countries to the European Union in 2004, subsequent financial support allowed the implementation of regular oral vaccination campaigns in the three countries since 2006 and ORV are still ongoing. This paper reviews ten years of surveillance efforts and oral vaccination campaigns conducted in the frame of European Commission programmes. Through the epidemiological analysis of rabies surveillance in these countries and an in-deep analysis of the ORV monitoring results, this paper emphasizes determinants of success and draws lessons for the future. These findings could provide valuable insights into the strategy required for rabies elimination and may help guide future implementation of oral vaccination programmes.

## Materials and Methods

### Study area

**Fig 1:**
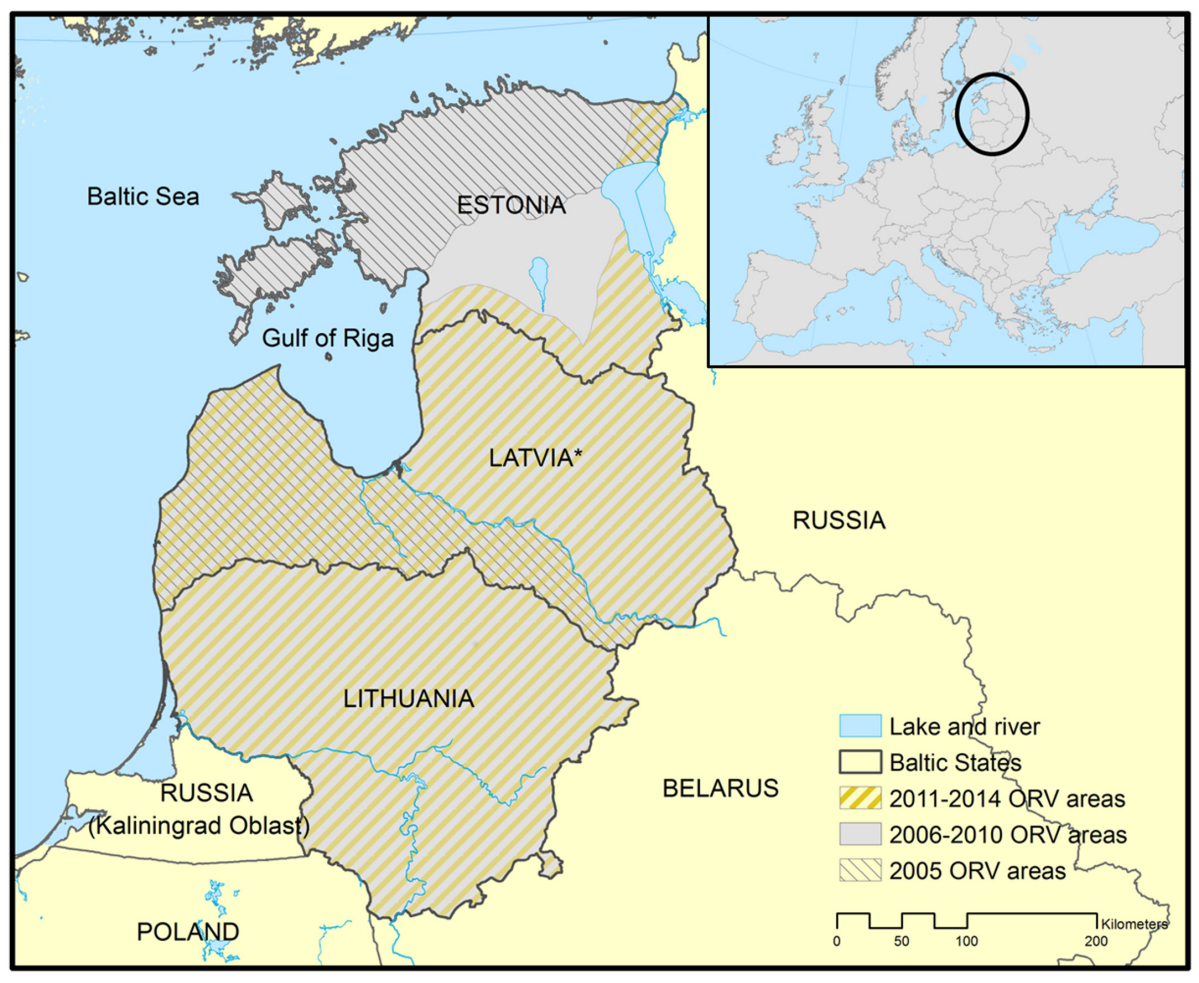
Location of the study and evolution of ORV areas. *Latvia: 2008 and autumn 2011: ORV not fully completed; spring 2014: ORV not carried out.

Covering approximately 175,000 km^2^, the Baltic States lie in the northeastern part of Europe and comprise the countries of Estonia (45,227 km^2^), Latvia (64,589 km^2^) and Lithuania (65,303 km^2^) (Fig 1). The Baltic States are bounded on the west and north by the Baltic Sea, which gives the region its name, on the east by Russia (511 km of common border), on the southeast by Belarus (818 km), and on the southwest by Poland (104 km) and an exclave of Russia named Kaliningrad (255 km). The topography of this area is relatively flat (culminating points in the three countries are around 300 m), characterized by numerous lakes and ponds, especially in the north, and hills in Lithuania. The most commonly encountered landscape is the temperate forest covering between 35 and 50% of the territories.

### Rabies surveillance

All suspect non-flying mammals exhibiting clinical signs suggestive of rabies or showing abnormal behaviour, animals found dead in the field including road kills and those to which humans have been exposed (bites, scratches, licking of wounds or contamination of mucous membranes with saliva) are defined as indicator animals and are submitted for diagnosis [19]. The sampling scheme focusing on these animals, covering the whole country territory, is herein defined by expert committees of the WHO (World Health Organization) and EFSA (European Food Safety Authority) as the surveillance system [2,19]. All collected samples were shipped and analyzed in the respective National Reference Laboratories of each Baltic country (Estonian Veterinary and Food Laboratory for Estonia; Institute of Food Safety, Animal Health and Environment "BIOR" for Latvia and National Food and Veterinary Risk Assessment Institute of Lithuania for Lithuania). Brain tissues were analyzed for viral antigens using the Fluorescent Anti-body Test (FAT) which is the gold standard technique for rabies diagnosis [22,23]. For all three countries, FAT-negative results of animals involved in human exposure and FAT-inconclusive results were confirmed using the rabies tissue culture infection test (RTCIT) [24], Reverse Transcription Polymerase Chain Reaction (RT-PCR) [25] or Real-Time Polymerase Chain Reaction (RT-qPCR) [26,27].

### Oral vaccination strategy

**Fig 2:**
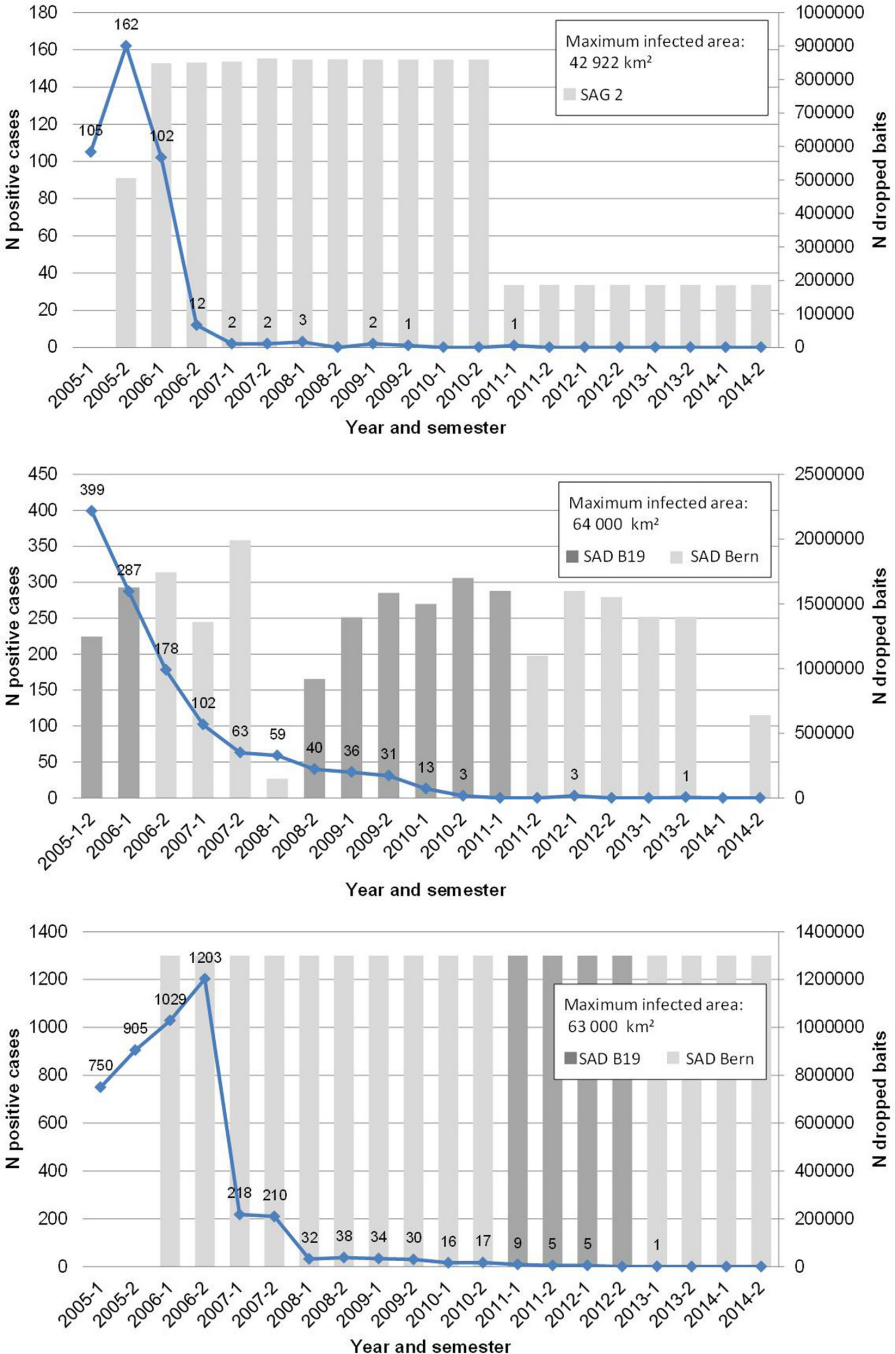
Evolution of the number of detected rabies cases over time, number of dropped baits and type of vaccine used in the three Baltic countries (top to bottom: Estonia, Latvia and Lithuania) since 2005.

The first wildlife ORV campaign in Estonia was organized in autumn 2005 and covered 57% of Estonian lands in the northern part of the country as part of a PHARE Twining Light Project (Fig 1) [16]. Vaccination programmes covering the entire territory (excluding urban areas, roads, water bodies and wet fields) representing approximately 43,000 km^2^ were carried out from 2006 to 2010. Bait distribution was performed twice a year, in spring (May, early June) and in autumn (September, October) as recommended by WHO and EFSA [2,19]. Baits were distributed at a rate of 20 baits per km^2^ using small fixed-wing aircraft flying at an altitude of 100–150 m, speeds of 150–200 km/h and in parallel flight lines (global positioning system (GPS) routes followed by the plane) distanced of 600 m [16,28]. Since spring 2011, ORV campaigns have been conducted only in a buffer zone of 9,325 km^2^ adjacent to neighbouring infected countries (Russia and Latvia) to ensure a sufficient level of immunity among raccoon dog and red fox populations. No automatic dropping device was used in the airplanes and no additional manual distribution was carried out in the field. A single vaccine bait type was selected through a tendering process, the modified live attenuated SAG2 vaccine (RABIGEN, Virbac Laboratories, France) (Fig 2).

In Latvia, following a PHARE Twinning Light project, ORV campaigns were carried out in 2005 for the first time via aircraft in half of the country (the size of vaccination area was 28,000 km^2^ and it was delimited by natural barriers) twice a year with 23 baits per km^2^. Starting from 2006, two vaccination campaigns were implemented in the entire territory (64,589 km^2^) (except in 2008 and autumn 2011 when ORV campaigns were incomplete and in spring 2014 where no ORV was carried out). Since 2006, between 21 and 31 baits per km^2^ have been distributed using flight line distances of 1000 m until 2008, 1000 m and 500 m alternately between 2008 and 2011, and 500 m since 2011. An automatic dropping device has been used since 2007 to distribute the baits. The type of vaccine purchased varied according to the procurement procedure. In general, two vaccines were used within the period 2005–2011—SAD B19 vaccine (FUCHSORAL, IDT Biologica GmbH, Germany) and SAD Bern (LYSVULPEN, Bioveta, Czech Republic). Since 2012, only the Lysvulpen vaccine has been in use (Fig 2).

In Lithuania, ORV programmes have been implemented since 2006 using aircrafts over the whole country (65,000 km^2^) except lakes, urban areas and the Ignalina nuclear power-station. The no-fly area surrounding the Ignalina power plant was covered by manual distribution of baits. Like in other countries, the vaccination strategy has been implemented biannually (one vaccination in spring between March and May and one vaccination in autumn between October and December). Parallel flight lines generally separated by 1000 m (since 2011, 500 m in areas on the Belarus border) at an altitude of 150–200 m and speed 150–200 km/h were used to distribute 20 baits per km^2^ [29] (Fig 2). Since 2006, only the Lysvuplen vaccines have been distributed except in 2011 and 2012 when Fuchsoral vaccines were used.

### Monitoring of ORV

In addition to the sampling scheme designed for rabies surveillance, a second sampling plan defined as monitoring of ORV was set up in vaccinated areas to evaluate the efficacy of ORV campaigns in terms of bait consumption (bait-uptake) and herd immunity [2,19,30]. This sampling focused on the collection of animals (red foxes and raccoon dogs) targeted by oral vaccines. These animals sampled by hunter associations are therefore considered as not suspected for rabies. Herd immunity level was assessed by enzyme-linked immunosorbent assays (ELISAs) [31]. Two commercial anti-rabies ELISA kit were used within the study: the BioPro ELISA (BioPro, Czech Republic) and the Platelia Rabies II kit (Bio-Rad, France). Their technologies differ by their coating aspect. The BioPro ELISA is a blocking ELISA using the crude glycoprotein to coat the plates and a positivity threshold (expressed as a percentage of blocking) of 40% [32,33]. The Platelia Rabies II kit is an indirect test using a purified rabies glycoprotein for the coating [34]. Serum titers were expressed as Equivalent Units per milliliter (EU/mL) with a cut-off of positivity fixed at 0.5 EU/mL in Estonia and Lithuania and 0.125 EU/mL in Latvia. The BioPro Rabies ELISA Ab kit was used in Latvia only.

Bait uptake was investigated by collecting red fox and raccoon dog jaws and by analysing the tetracycline (TTC) specific fluorescence in thin tooth sections under ultraviolet light [35,36]. Indeed, after its inclusion in the coating of the bait and its consumption by the targeted animal, the tetracycline molecule, used as a bait uptake marker, is incorporated into bones and teeth. This interaction creates a line that can be detected using epi-fluorescence microscopy. Each animal sampled for monitoring were analyzed for both serological analysis and tetracycline detection when possible (depending of the organs let intact by the shot of the hunter).

Studied animals from surveillance and monitoring scheme were originated from the field, died of natural causes and during the hunting/vaccination program developed and launched by the ministry of each country. These sampling processes were realised in compliance with the legislation of each country and under the recommendations of international institution (WHO [2] and EFSA [19]). In Europe, such process does not require any specific ethical approval as animals are received only dead in laboratories. Hunting plans are organised in the frame of control programmes of the disease and organised by Member States.

### Phylogenetic analysis of rabies cases

A panel of 165 field rabies viruses was collected in Baltic countries between 2004 and 2013 for this study. The isolates investigated from domestic and wild animals were extracted from brains of animals samples in Estonia (n = 43), Latvia (n = 42) and Lithuania (n = 80). The samples were isolated from 12 different wild and domestic animal species: *Nyctereutes procyonoides* (65), *Vulpes vulpes* (44), *Canis familiaris* (14), *Bos taurus* (11), *Felis catus* (ten), *Procyon lotor* (four), *Meles meles* (three), *Equus caballus* (two), *Martes martes* (two), *Ovis aries* (one), *Canis* (one), *Lynx lynx* (one) and six non-determined species (S1 Table). The samples were initially tested using the FAT prior to genetic characterization [23].

### Viral RNA extraction

#### Estonian isolates

Ten percent (w/v) brain homogenates were prepared for ribonucleic acid (RNA) extraction using dulbecco's modified eagle's medium (DMEM) medium and centrifuged at 1,500 g for ten minutes for clarification. Viral RNA was extracted from 200 μL supernatant using an Iprep PureLink Virus kit (Invitrogen, France) according to the manufacturer’s instructions and stored at -70°C until use.

#### Latvian isolates

Ten percent (w/v) brain homogenates were prepared for RNA extraction using phosphate-buffered saline (PBS) medium and centrifuged at 2,500 rpm for 30 min for clarification.

Viral RNA was extracted from 140 μL supernatant using the RNeasy Mini Kit (Qiagen, Germany) according to the manufacturer’s instructions. RNA was eluted with 70 μL of RNAse—free water and used immediately for RT-PCR. The remaining RNA was stored at -80°C for further investigation.

#### Lithuanian isolates

Five percent brain homogenates were prepared using RLT lysis buffer (Qiagen, Lithuania) and centrifuged for 3 minutes at 13,200 g for clarification. RNA was extracted directly from the lysate using the RNeasy Mini Kit (Qiagen, Lithuania) according to the manufacturer’s instructions. RNA was eluted with 70 μL of RNAse—free water and used immediately for RT-PCR. The remaining RNA was stored at -20°C for further investigation.

### HnRT-PCR and sequencing

#### Estonian isolates

Viral RNA extracted from the ten percent brain homogenate supernatant was used for partial nucleoprotein (N) gene amplification (positions 55–660 compared with the pasteur virus (PV) strain (GenBank: NC001542) as previously described [37]. The conserved sequence from the N gene was amplified with primers JW12 (forward: 5’-ATGTAACACCYCTACAATG) and JW6 (reverse: 5’-CARTTVGCRCACATYTTRTG) in the first round of PCR (polymerase chain reaction) and JW12 and JW10 (forward: mix of 5’-GTCATCAAAGTGTGRTGCTC, 5’-GTC ATCAATGTGTGRTGTTC and 5’-GTCATTAGAGTATGGTGTTC) in the second PCR. Following amplification, the PCR products (589-bp) were separated by electrophoresis on a two percent agarose gel and purified with a commercial kit (Nucleospin Extract II columns, Macherey Nagel, France) according to the manufacturer’s instructions. Gel purified PCR products were sequenced in both directions by Beckman Coulters Genomics (Takeley, Essex, United Kingdom) with the same specific primers used for the nested PCR amplification.

#### Latvian isolates

Viral RNA extracted from the ten percent brain homogenate supernatant was amplified with primers JW12-JW6 in the first PCR and with JW12-JW10 in the second PCR, as previously described [37]. Specific amplification of the glycoprotein (G) gene was performed on the badger (*Meles meles*) isolate numbered DR-784 (S1 Table) isolated in Latvia in 2013. One-step RT-PCR amplification was performed with primers GH3 (positions 3891–3908) and GH4 (positions 4621–4602), as previously described in Bourhy et al. [38]. Cycling conditions were identical to the N gene amplification except for the annealing temperature (55°C for N gene and 61°C for G gene). The PCR products (589-bp and 710-bp) were analyzed on a 1.8% agarose gel. The amplified PCR products were purified using 500 U of SAP (Shrimp Alkaline Phosphatase, Fermentas, Lithuania) and 10 U of ExoI (Exonuclease I, Fermentas, Lithuania) by incubation at 37°C for 15 min, followed by an inactivation of enzymes at 85°C for 15 min. Sequencing was performed using BigDye Terminator v3.1 Cycle Sequencing Kit (Applied Biosystems, USA) according to the manufacturer's protocol.

#### Lithuanian isolates

Five μl of RNA was mixed with 0.8 μM each of forward (JW12) and reverse (JW6) primers in 25 μl of RT-PCR solution containing 1x Qiagen One step RT-PCR buffer, 0.4 mM each deoxyribonucleotide triphosphates (dNTP) and 0.04% (v/v) one step RT-PCR enzyme mix (Qiagen) as described in Orlowska et al. [39]. Amplifications were performed using the following programme: one cycle of RT at 50°C for 30 min, followed by denaturation for 15 min at 95°C, 35 amplification cycles with denaturation at 94°C for 30 sec, annealing for 30 sec at 49°C and extension for 1 min at 72°C, and a final extension at 72°C for 10 minutes. PCR products were analyzed on a 1.5% agarose gel. The amplified PCR products were purified using the same protocol that previously described for Latvian isolates. Sequencing was performed using BigDye Terminator v3.1 Cycle Sequencing Kit (Applied Biosystems, Lithuania) according to the manufacturer's protocol.

All necessary safety measures were followed to prevent any cross-contamination and false positive results when performing PCRs [40].

### Nucleotide sequence analysis and phylogenetics

For all the samples, forward and reverse sequences were assembled and edited using the ContigExpress program of Vector NTI software, version 11.5.3. (Invitrogen, France). Alignments were edited using Genedoc software, version 2.7.000 [41]. The same software was used to translate the gene sequence. Percentage identities and similarity scores were determined using the BIOEDIT program version 7.2.5. [42]. After the alignment of sequenced amplified PCR products, 106 identical sequences (56 from Lithuania, 23 from Latvia and 27 from Estonia) showing 100% nucleotide identity for the N gene (460 nt) were removed from the phylogeny. Fifty-nine partial N gene sequences (24 from Lithuania, 19 from Latvia and 16 from Estonia) were available for subsequent analysis. The dataset contained 93 sequences (361 nucleotides, positions 109 to 470 compared with the challenge virus standard (CVS)-11 strain GenBank no. GQ918139). Fifty nine representative Baltic samples, eight isolates from neighbouring countries (six from Poland and two from Russia), two from Ukraine, seven from Europe, two fixed strains (D42112 and HQ829841), three representatives of rabies vaccine strains (EF206708, EF206709 and EF206719), six referenced Artic and Artic-like isolates and six reference strains used as outgroup were included in the dataset (S1 Table).

### Statistical analysis

#### Surveillance and ORV monitoring data analysis

Generalized linear models (GLMs) including the matrix “number of positive samples and number of negative samples” as response variable were used to study the variations in vaccination coverage after ORV in red fox and raccoon dog populations. Both herd immunity (samples positive for rabies antibody content) and bait uptake analysis (samples with presence of tetracycline in teeth) were therefore analysed using logistic regression. The explanatory variables included the factor “SPECIES” (red foxes vs raccoon dogs), “SEASON” (spring campaign vs autumn campaign), “YEAR” (as continuous variable) and “COUNTRY” (Estonia vs Latvia, vs Lithuania). Because the variable “AGE” (juvenile vs adult) of raccoon dogs and red foxes was available in the Estonian and Lithuanian dataset for TTC analysis, and in the Lithuanian dataset for the serological analysis, we performed the analysis including the variable “AGE” in a second step, on Lithuanian and Estonian data only. The variable “BAIT DENSITY” (number of baits dropped per km^2^ in the vaccinated area) and “BAIT TYPE” (Lysvulpen vs Fuchsoral) could be assessed on the Latvian dataset because it was the only country in which two types of bait were alternatively used and where bait densities varied (in contrast, 20 baits per km^2^ were used continuously in Lithuania and Estonia). Considering the serological data of collected animals, Latvia was the only country having used two kinds of ELISA kits (ELISA BioPro vs ELISA Platelia II) (variable “KIT”). A third analysis restricted to the Latvian data was consequently conducted to explore the potential influence of these technical factors on ORV monitoring. These analyses included also the variable “SPECIES”, “SEASON” and “YEAR”.

For each of the three GLM analyses, models of all possible combinations of variables were compared using the information-theoretic method corrected for small samples size outlined by Burnham and Anderson [43]. Differences in the Akaike second-odrer information criterion (AICc) between the best model and all other considered models were calculated to determine the relative ranking of each possible model. The model with the lowest AICc represented the best compromise between the residual deviance and number of variables. When ΔI was inferior to two (Δi = difference between AICc and the lowest AICc value), the most parsimonious model (i.e., that with the fewest variables) was selected. We tested for a significant difference between specific variables using the Wald test [43]. Goodness-of-fit of the selected model was assessed by graphic exploration in addition with a chi-square test based on the residual deviance and degrees of freedom. When over-dispersion was diagnosed in the model validation process, we performed and *ad hoc* adjustment using the approach discussed by Williams (1982). Over-dispersed binomial logit models were consequently estimate using a quasi-binomial distribution (quasi-likelihood approach). Odds ratio of each variable and their interval confidence were computed by calculating the exponential of the corresponding coefficient parameter.

The computing of 95% confidence intervals (95% CI) of proportions (proportion of bait uptake and herd immunity), graphics and GLMs were performed using the R open-source software, version 3.0.1 [44].

#### Phylogenetic analysis

Phylogenetic analysis was performed using the maximum likelihood (ML) method with the software SeaView and PhyML, version 3.1. [45]. ML analysis was undertaken using the GTR+ γ (General-Time Reversible model using a gamma-shaped distribution of rates across sites) model with a Log likelihood of the current tree (Lk) of -2261.94. The robustness of tree topology was calculated using 100 replicates. Bootstrap values over 70% indicated significant support for the tree topology [46]. The tree’s graphic representation was constructed with Fig Tree software, version 1.4.2 (http://tree.bio.ed.ac.uk/software/figtree/). Deduced amino acid sequences were used to compare the variation in amino acid sequences among Baltic sequences.

In addition, a Bayesian analysis of the Baltic nucleotide sequences including the same dataset of 93 sequences was performed using the general-time reversible model (GTR) model of the TOPALI version 2.5 graphical interface (http://www.topali.org/) [47]. The model used was: GTR+γ, 10^7^ generations, number of runs of 4, sample frequency of 1000 and burn-in of 25%. The phylogenetic tree was visualised by using FigTree (version 1.4.1; http://beast.bio.ed.ac.uk/FigTree).

## Results

### Rabies surveillance

A total of 24,919 animals were diagnosed for rabies from 2005 to 2014 in the Baltics. Around 70 to 80% of all detected positive cases were found in red foxes and raccoon dogs (For Estonia, 35% foxes and 48% racoon dogs; for Latvia, 40% foxes and 30% raccoon dogs; for Lithuania 31% foxes and 40% raccoon dogs). In the three countries, the maximum number of detected rabies cases was observed during the 2005–2006 period (Fig 2). The highest number of detected cases was recorded at the same semester of the implantation of the first ORV in Estonia and Latvia, while one semester after the first ORV in Lithuania. The ORV induced indisputably a decrease of the number of positives cases in the three countries (excepted in Lithuania between the second semester of 2006 and the first semester of 2007). Regarding the maximum number of cases observed in each country, 90% reduction of detected cases was reached after two ORV campaigns in Estonia, eight in Latvia and four in Lithuania. The last rabies case (field strain) was notified in 2011 in Estonia, in 2012 in Latvia and in 2013 in Lithuania. When starting ORV, surveillance effort (number of indicator animals sampled per 100 km^2^ in the whole country) ranged from 1.7 to 1.3 in Estonia (2005 and 2006), 1.7 to 1.6 in Latvia (2005 and 2006) and 5.8 in Lithuania (but caution must be taken in the interpretation of this number because animals collected for monitoring were also included for this latter country). Since no rabies cases were detected, the pressure of surveillance appeared also comparable between the three countries ranging from 0.42–0.30 in Estonia (2012–2014), 0.42–0.30 in Latvia (2013–2014) and 0.48 in Lithuania (2014). These data thus support the comparability of the number of positive cases in the different countries in recent years.

### Monitoring of ORV

#### Bait uptake

**Fig 3:**
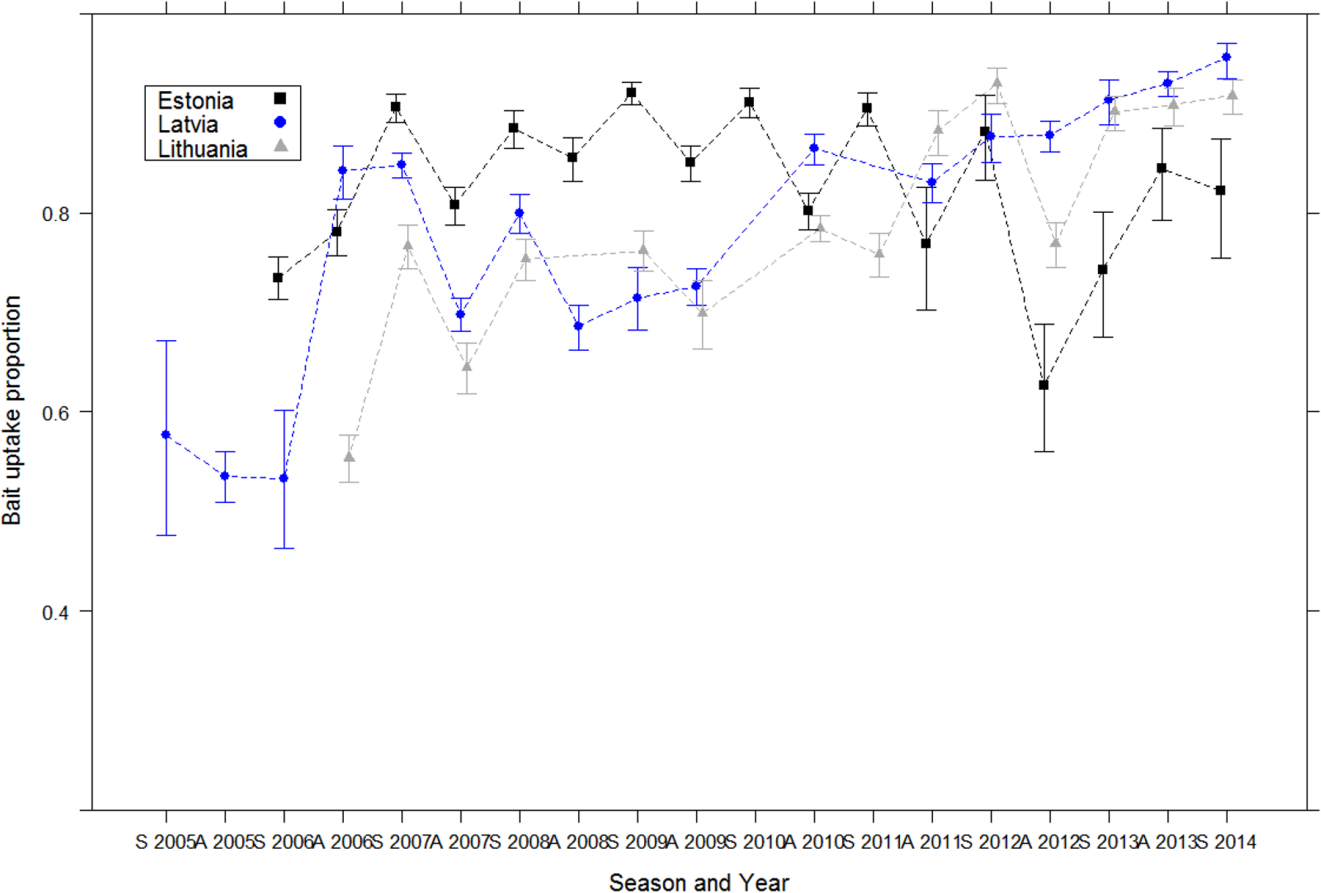
Evolution of the bait uptake in the three Baltic countries (red fox and raccoon dog data) after implementation of ORV EU programmes in the Baltic States. (S: spring; A: autumn).

A total of 64,989 animals were analyzed for tetracycline presence in teeth. The overall average bait uptake was 80%. Fig 3 shows a slight general upward trend in bait uptake during the whole study period (2005–2014) in Latvia and Lithuania. In Estonia, bait uptake strongly increased from spring 2006 to spring 2007 and remained stable until autumn 2012.

**Fig 4:**
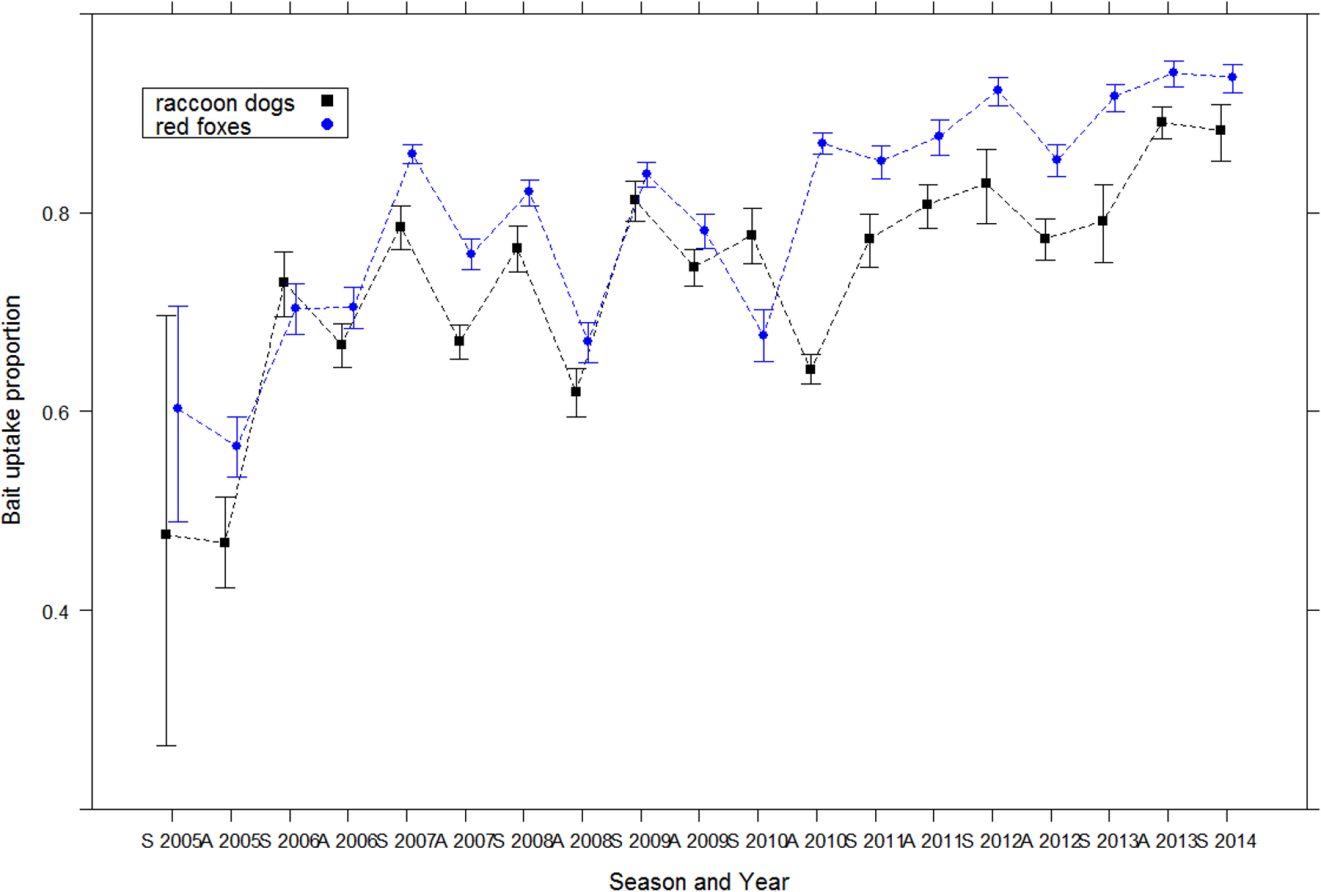
Evolution of the bait uptake over time in red foxes and raccoon dogs in the Baltic States.

The comparison of all model combinations showed that the model including the variables “COUNTRY”, “SPECIES”, ‘SEASON” and “YEAR” was the model that best fit the dataset (AICc = 3994.4; Akaike weight = 1). As the binomial data were over-dispersed, selected GLM was then fitted using quasi-binomial function. Odds ratio of the model indicated that the proportion of positive TTC samples was significantly lower in raccoon dogs than in red foxes (Fig 4), increased over years and was lower in Lithuania than in Estonia (Table 1A).

**Table 1:** Variables associated with the bait uptake and corresponding odds ratio of the selected model for the Baltic States data (a), for the Estonian and Lithuanian data with the variable “AGE” (b) and for the Latvian data with the variables “BAIT DENSITY” and “BAIT TYPE” (c).

Variable	Group	N pos (%)	OR	CI OR(95%)	P-value (Wald test)
		a) Baltic States			
**SPECIES**	Red fox	32,406 (83)	REF		
	**Raccoon dog**	18,693 (74)	0.61	0.458 ; 0.810	**0.0007**
SEASON	Autumn	37,414 (75)	REF		
	Spring	23,685(83)	1.16	0.876 ; 1.550	**0.3**
**COUNTRY**	Estonia	15,643 (85)	REF		
	Latvia	19,083 (78)	0.70	0.483 ; 0.997	0.05
	**Lithuania**	16,373 (74)	0.46	0.318 ; 0.650	**<0.0001**
**YEAR**			1.17	1.101; 1.236	**<0.0001**
		b) Estonia and Lithuania						
**AGE**	Adult	22,537 (79)	REF		
	**Juvenile**	9,477 (78)	0.58	0.431 ; 0.774	**0.0002**
**SPECIES**	Red fox	19,291 (82)	REF		
	**Raccoon dog**	12,723 (74)	0.67	0.500 ; 0.897	**0.0007**
SEASON	Autumn	14,904 (75)	REF		
	Spring	17,110 (83)	1.28	0.952; 1.713	**0.1**
**COUNTRY**	Estonia	15,643 (85)	REF		
	**Lithuania**	16,371 (74)	0.49	0.361 ; 0.653	**<0.0001**
**YEAR**			1.13	1.067; 1.207	**<0.0001**
		c) Latvia						
**SPECIES**	Red fox	13,114 (81)	REF		
	**Raccoon dog**	5,969 (74)	0.53	0.404 ; 0.696	**<0.0001**
**BAIT**	Fuchsoral	6,130 (70)	REF		
	**Lysvulpen**	12,953 (83)	2.33	1.743 ; 3.142	**<0.0001**
SEASON	Autumn	12,509 (76)	REF		
	Spring	6,574 (83)	0.84	0.604 ; 1.157	0.28
DENSITY			0.96	0.910 ; 1.011	0.13
**YEAR**			0.16	1.109 ; 1.242	**<0.0001**

Significant factors are shown in bold. OR: odds ratio. CI OR: Confidence Interval of the odds ratio. N pos: Number of positive samples. REF: Referent factor.

For Estonia and Lithuania where the variable "AGE" was considered, the best model included the variables “AGE”, “SEASON”, “SPECIES”, “YEAR” and “COUNTRY” (AICc = 3763.0, Akaike weight = 1). Because the binomial data were over-dispersed, selected GLM was fitted using quasi-binomial function. Odds ratio of the selected model indicated that juvenile animals harboured a lower frequency of TTC in teeth than adults (Table 1B). Conclusions on the effect of “YEAR”, “SPECIES” and “COUNTRY” remained the same as in the previous global analysis. For both analyse, graphic explorations and the Pearson goodness-of-fit test indicated that the models fit the data well (P = 0.48 and P = 0.48 respectively).

**Fig 5:**
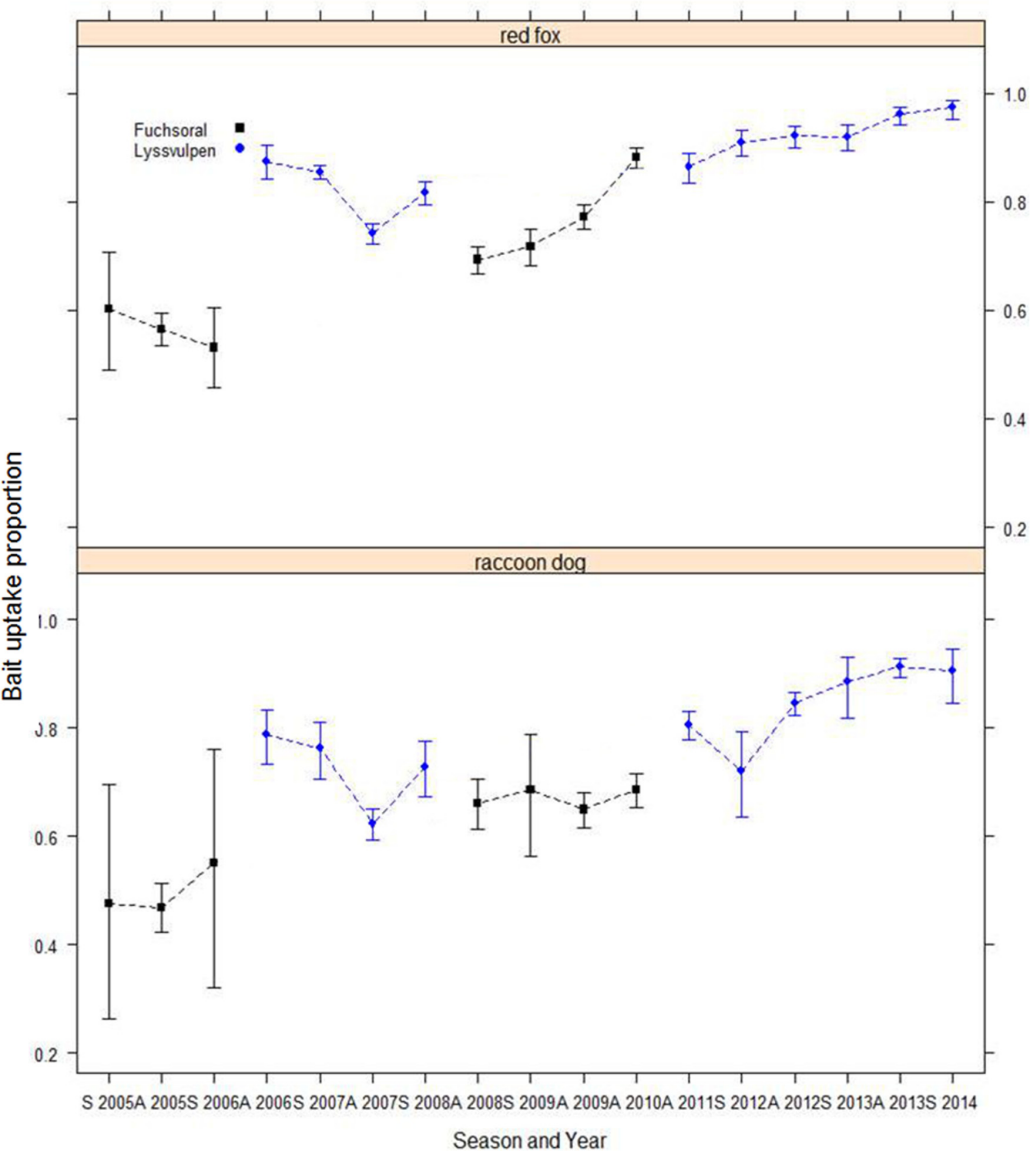
Evolution of the bait uptake in Latvia in red foxes and raccoon dogs using two different bait types.

For Latvia where the variables “BAIT DENSITY” and “BAIT TYPE” were also considered, the final selected model included all the variable, of which “BAIT DENSITY” and “BAIT TYPE” (AICc = 712.3, Akaike weight = 0.996). As for previous analysis, data being over-dispersed, the selected GLM was fitted using quasi-binomial function. Odds ratio of the selected model indicated that the bait uptake increased over years, that raccoon dogs were less frequently marked than red foxes and that the frequency of TTC positive samples was two times higher when the type of bait used was Lysvulpen (Table 1C and Fig 5). Graphic explorations and the Pearson goodness-of-fit test indicated that the models fit the data well (P = 0.47).

#### Seroconversion

**Fig 6:**
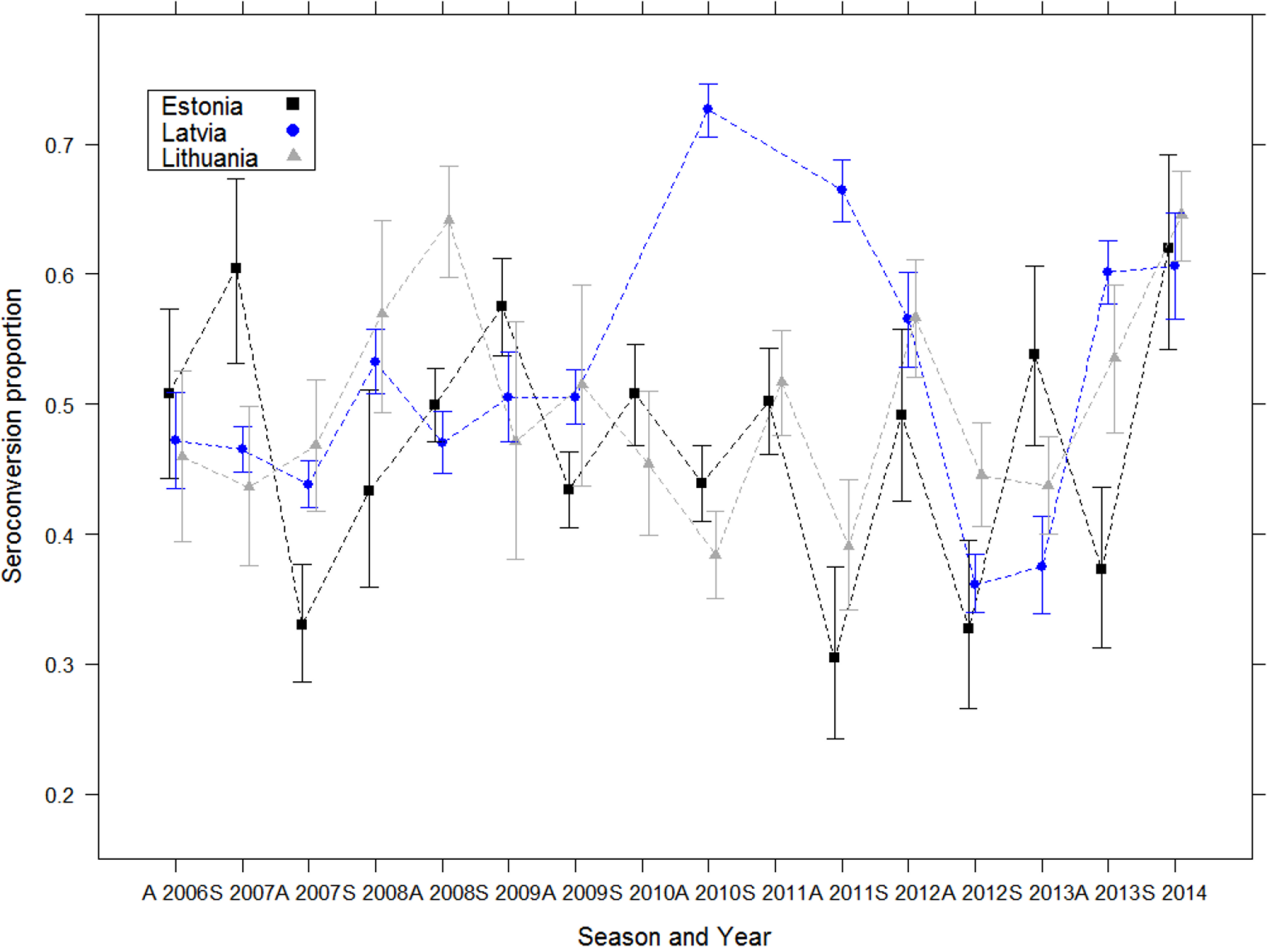
Evolution of the seroconversion level in the three Baltic countries (red fox and raccoon dog data) after implementation of ORV EU programmes in the Baltic States.

The seroconversion level in the target population (I.e. red foxes and raccoon dogs) was assessed after each ORV and on a total of 36,948 individuals. No influence of the length of time since the first ORV was observed on the herd immunity level (Fig 6). Globally (independently from the country and the year), the percentage was approximately 50%. Maximum seroconversion level reached 73% in autumn 2010 in Latvia whereas the minimum observed level was 30% in autumn 2011 in Estonia.

The selected logistic model included all the variables (AICc = 2112.0, Akaike weight = 0.448). Odds ratio of the quasi-binomial model indicated no significant difference in seroconversion level whatever the considered variable (Table 2A). Graphic explorations and the Pearson goodness-of-fit test indicated that the models fit the data well (P = 0.48).

**Table 2:** Variables associated with the seroconversion and corresponding odds ratio included in the selected model for the three Baltic States data (a), for the Lithuanian data with the variable “AGE” (b) and for the Latvian data with the variable “BAIT DENSITY”, “BAIT TYPE” and “KIT” (c).

Variable	Group	Npos (%)	OR	CI OR (95%)	P-value(Wald test)
		a. Baltic States			
SPECIES	Red fox	11, 666 (51)	REF		
	Raccoon dog	6,876 (49)	1.02	0.852; 1.229	0.81
SEASON	Autumn	11,414 (50)	REF		
	Spring	7,128 (51)	1.17	0.971; 1.416	0.10
COUNTRY	Estonia	3,647 (47)	REF		
	Latvia	11,482 (51)	1.24	0.992; 1.556	0.06
	Lithuania	3,413 (50)	1.04	0.834; 1.304	0.71
YEAR			1.01	0.973; 1.053	0.55
		b. Lithuania			
SPECIES	Red fox	2,560 (50)	REF		
	Raccoon dog	851 (47)	0.97	0.730; 1.275	0.8
SEASON	Autumn	1,612 (47)	REF		
	Spring	1,799 (53)	1.09	0.825; 1.436	0.5
AGE	Adult	3,102 (50)	REF		
	Juvenile	309 (42)	0.76	0.569;1.021	0.07
		c. Latvia			
SPECIES	Red fox	7,684 (52)	REF		
	Raccoon dog	3,798 (51)	0.98	0.792 ; 1.210	0.85
SEASON	Autumn	7,706 (52)	REF		
	Spring	3,776 (50)	0.85	0.647 ; 1.117	0.25
BAIT	Fuchsoral	3,788 (56)	REF		
	Lysvulpen	7,694 (50)	0.90	0.698 ; 1.157	0.41
**KIT**	Biopro	916 (37)	REF		
	Platelia II	10,566 (53)	2.85	2.013 ; 4.065	**<0.0001**
DENSITY			0.99	0.953 ; 1.033	0.74
**YEAR**			1.12	1.071 ; 1.180	**<0.0001**

Significant variables are shown in bold. OR: odds ratio. CI OR: Confidence Interval of the odds ratio. N pos: Number of positive samples. REF: Referent factor.

The same analysis was performed with the addition of the factor “AGE” for the Lithuanian data. The best selected logistic model included all the variable excepted the variable “YEAR” (AICc = 739.4, Akaike weight = 0.379). No significant difference of odds ratios were observed in the quasi-binomial model, whatever the variable considered (Table 2B). Both graphic explorations and Pearson goodness-of-fit test indicated that the models fit the data well (P = 0.48).

**Fig 7:**
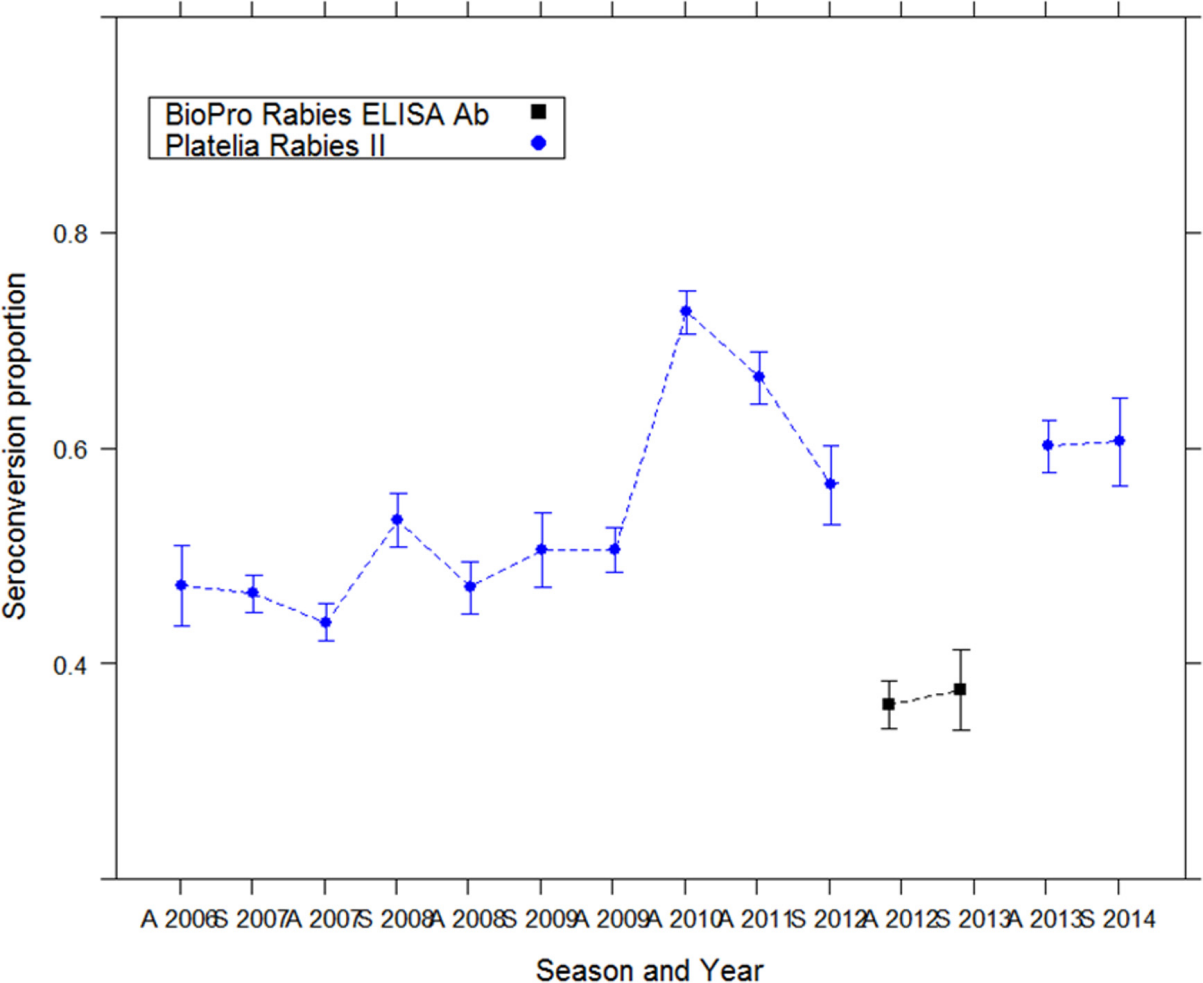
Evolution of the seroconversion in Latvia (red fox and raccoon dog data) analysed using two different ELISA kits.

The third serological analysis focused on the technique used in Latvia (ELISA kit and BAIT type, both of which varied in the Latvian rabies eradication program). The selected logistic model included all the variables (of which “KIT” and “BAIT”) (AICc = 559.9, Akaike weight = 0.481). Odds ratio of the selected model indicated that the Bio-Rad kit was associated with higher seroconversion levels (3 times higher when using Platelia 2 from Biorad compared to the BioPro test) and that seroconversion increased over year (Table 2C and Fig 7). Graphic explorations and the Pearson goodness-of-fit test indicated that the model fit the data well (P = 0.46).

### Phylogenetic analysis

**Fig 8:**
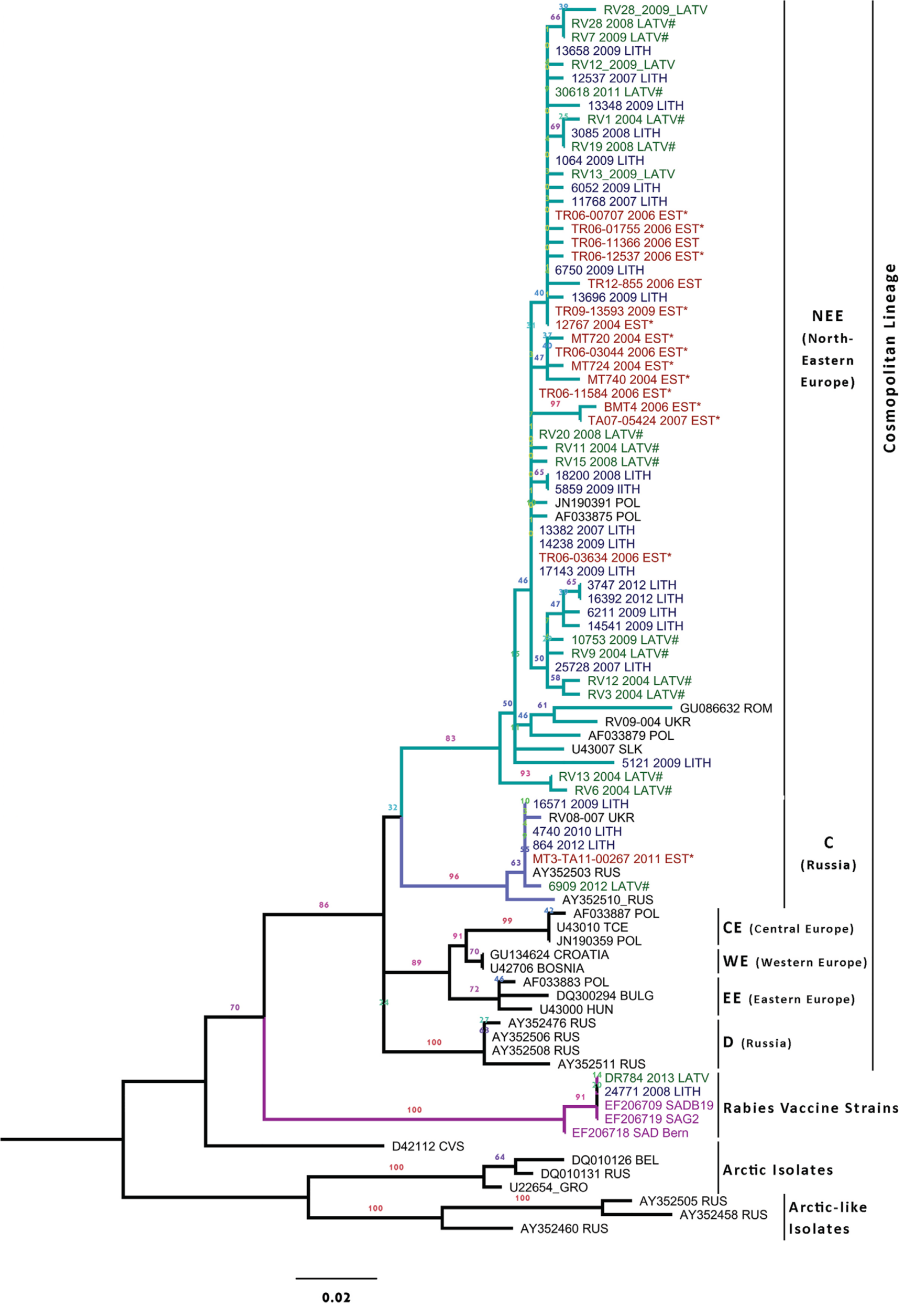
PhyML phylogenetic tree of the 59 representative isolates from Baltic States included in the study. Abbreviations for the previous described European (Central Europe (CE), Eastern Europe (EE), North-East Europe (NEE) and Western Europe (WE)) and Russian (C) groups are given according to Bourhy et al. [38] and Kuzmin et al. [48].

The two phylogenetic analyses of the partial N gene sequences performed using either PhyML or Mr Bayes produced trees with identical topology. The phylogenetic analysis showed that 163 of the 165 studied Baltic sequences belonged to the lineage formed by the classical rabies virus within the cosmopolitan lineage, with a bootstrap value of 86% (Fig 8). No Arctic or Arctic-like variants were identified in the panel of viruses studied from the Baltic States.

The majority of the Baltic rabies isolates grouped with the North-East European lineage (NEE), forming one strongly supported group (bootstrap value of 83). The NEE group consisted of 52 samples from the Baltic States and 21 published viral sequences (S1 Table) [38,49–51]. Both wild and domestic species fell in the NEE group.

The NEE Group showed less than 1% nucleotide divergence and 3% amino acid divergence among all Baltic isolates. Nucleotide sequence analysis showed 100% of nucleotide identity between a red fox sample (no. 11584) isolated in 2006 in Estonia and three samples from Lithuania (a red fox isolated in 2007, a raccoon dog and a cattle both isolated in 2009). The same perfect identity was obtained between the Estonian isolate no. 11584 and two samples from Latvia (a raccoon dog and a dog both isolated in 2008).

Five sequences from the Baltic States clustered with C group [48] formed with two published sequences, one from Russia and one from Ukraine (bootstrap support of 96%). Four species were included in this group: red fox (n = 2), raccoon dog (n = 1), cattle (n = 1) and dog (n = 1). Within C group, sequences showed more than 99.9% of nucleotide similarity. 100% nucleotide identity was shown between a red fox sample (MT3-TA11-00267) isolated in Estonia in 2011 and two samples from Lithuania; a dog (no. 864) in 2012 and a raccoon dog (no. 4740) isolated in 2010.

The PhyML tree also showed that a badger (*Meles meles*) (no. DR 784) and a marten (*Martes martes*) (no. 24771) isolated in Latvia in 2013 and in Lithuania in 2008 respectively, belonged to the group formed by the rabies virus vaccines (bootstrap of 100) (Fig 8). The vaccine-induced case isolated in Lithuania was found in the Alytus district in the south of the country, an area vaccinated since 2006 with Lysvulpen baits, whereas the vaccine-induced case isolated in Latvia was found in the Aloja district, in the north of the country, an area also vaccinated with Lysvulpen baits since 2011. Nucleotide analysis of the partial N gene sequenced of the two isolates showed 100% of nucleotide identity with the two referenced SAD-derived laboratory vaccine virus strains (EF206719 and EF206709) and there was 99.4% nucleotide similarity with the SAD Bern vaccine strain (EF206708). The case found in Latvia was located in an area vaccinated with the Lysvulpen baits 25 km away from Estonia where the Rabigen baits were used. As the partial N gene sequence analysis did not discriminate among the two SAD-derived laboratory vaccine virus strains, the amplification of partial G gene sequence on the DR784 isolate was undertaken to identify the vaccine strain. The comparison between DR784 isolate and three vaccine strains, SAD B19 (EF206709), SAG2 (EF206719) and SAD Bern (EF206708), showed 100% nucleotide identity with SAD B19 and 99.8% of similarity with the SAG2 and SAD Bern vaccines. The isolate DR784 was characterized by the presence of arginine in codon 333 (G gene). The sequence was clearly distinct from SAG2 (EF206719), characterized by two mutations in codon 333 yielding glutamine (Gln) at this position instead of arginine (Arg).

## Discussion

### Importance of surveillance

Surveillance data indicated a drastic reduction (90%) in the number of detected cases following 1 to 4 years of ORV. These results corroborate those from other European countries where 90% reduction of rabies detected cases were observed within 10 years, and in many cases less than 5 years following first ORV [52–54]. Depending on the country, the time to complete elimination (i.e. remaining 10%) is more or less longer to achieve. While eradication requires an additional 10 or more campaigns until no more cases are detected in Freuling at al. [10] we found that 2 to 8 campaigns were necessary. Variation in the reduction of the number of cases detected after each ORV depends on multiple factors such as the geographical location of the infected country, the initial epidemiological situation, the tools and strategy used in the control programmes and indubitably the implementation of an appropriate surveillance scheme.

As soon as ORV was implemented on the whole territories, the proportion of positive cases started to decrease in the three countries. As suggested previously in Brochier et al. [55], and Aubert [56] for fox rabies and Townsend et al. [57] for dog rabies, an inadequate vaccination area can compromise success and considerably extend the time to elimination. For Lithuania, the animals collected in vaccinated areas for the monitoring of ORV were also diagnosed for rabies. Because this sampling focuses on the animal population targeted by oral vaccines and not suspected for rabies (in contrary to classic rabies surveillance plans where only suspect animals are collected), Lithuanian surveillance data probably overestimates the number of negative samples compare to other countries. The comparison of the percentage of positive cases between countries became consequently hazardous. For this reason, combining data issued from surveillance sampling and monitoring sampling should, insofar as possible, be avoided [2,22]. Appropriate surveillance schemes focus on indicator animals collected at anytime, anywhere throughout the country and no sample size can be defined for proving the absence or the presence of rabies in wildlife regardless of the reservoir species. In contrast, the monitoring schemes are based on sampling foxes and raccoon dogs shot by hunters in vaccinated areas after ORV campaigns [30].

### Implication of the presence of two important reservoirs

The oral vaccines used at the present time in Europe for raccoon dogs were developed to control rabies specifically in foxes. An experimental study evaluated the safety and efficacy of SAG 2 baits on caged raccoon dogs [58]. Either direct instillation or bait ingestion using a virus dose containing at least 10 times the field vaccine dose proved vaccine safety during the 60 days of observation of animals. More than 6 months after oral vaccination with the field dose, all animals were challenged with a street rabies virus. All vaccinated animals developed high rabies neutralizing antibody titers and survived a virulent challenge, demonstrating the effectiveness of the vaccine bait according to the European Pharmacopeia monograph. These results suggest that SAG2 vaccine baits are suitable for this species. Another study conducted on the SADP5/88 vaccine (derived from SAD Bern and no longer in use) in which two different doses of the vaccine were administrated showed satisfactory protection of challenged animals [59]. Paradoxically, to our knowledge, there are very few experimental studies using vaccines used in Baltic countries on raccoon dogs to assess their efficacy and safety prior to their release in the field.

For the first time, bait uptake results suggest a significant difference in the frequency of uptake of red foxes and raccoon dogs, with a lower proportion of tetracycline-positive raccoon dogs compared with red foxes. This result can be attributed to the difference in behavior of the two species and particularly to the hibernation of raccoon dogs in the Baltics during the cold season (November–March) [60], which may influence the epidemiology of the disease and access to vaccines distributed during this period. The impact of hibernation was suggested in a model of rabies transmission in both raccoon dogs and red foxes [61]. As suggested by our results, strategies to control rabies in countries where this species is an important transmitter should better focus on the raccoon dog biology. As example, ORV could also target raccoon dogs after they emerge from hibernation.

### Other factors affecting bait uptake

All countries implemented ORV according to the EU recommendations[19]. Bait uptake levels in Baltic countries rapidly reached 80% of the target population. Our study showed a constantly increasing bait uptake throughout the study period, suggesting cumulative exposure to distributed baits [19]. Data analysis in Estonia and Lithuania confirmed previous studies, showing a significantly lower bait uptake in juveniles than in adults [28,62,63]. As a matter of fact, difficulties in reaching juveniles during ORV campaigns were observed. This was observed especially in early spring [19] because cubs are in dens or cannot be vaccinated because too young to eat the bait. Latvia has used two types of vaccines, Lysvulpen and Fuchsoral vaccines. Analysis of factors that potentially affect bait uptake showed a significant influence of the type of bait used, with higher bait uptake when the vaccine Lysvuplen was used. The type of bait influence was independent from the year as further analysis, omitting the first years of vaccination with Fuchsoral baits, still considered the bait type as a significant variable explaining the TTC variations. Given that, according to the manufacturer’s specifications, both vaccines contain 150 mg of tetracycline in the bait matrix, the reason for this difference is unknown. More investigations on bait matrix composition and palatability are needed.

### Factors affecting the seroconversion

Neutralizing antibodies are the most reliable parameter for assessing the efficacy of vaccination because they are closely correlated with protection against rabies infection [64]. The assessment of the rabies antibody level is theoretically the best means for evaluating vaccination coverage because individual variation in immune reactions is taken into account. ELISAs allow large-scale screening because they are rapid, easy to perform, do not require live rabies virus or cell culture, and can be performed in any laboratory. These tests have been demonstrated as particularly suitable for assessing the effectiveness of oral vaccination in field samples [31,65,66]. The evolution of herd immunity level did not show any specific pattern, showing an unsteady evolution in all three countries. The surprising absence of any immunological trend may reflect the lack of sensitivity or reliability of some commercial ELISA kits, as has already been demonstrated recently [34,67,68]. Although the overall average bait uptake in this study was 80%, seroconversion level was approximately 50%. The same large discrepancies observed between uptake and seroconverion were attributed the lack of sensitivity of a commercial kit on field samples compared to blood samples taken from experimentally infected foxes and raccoon dogs, probably due to the reduced quality of the sera (haemolysis, bacterial contamination due to field condition) [28,67,69].

Latvia was the only country that used two different kinds of ELISA kits (Bio-Rad and BioPro) to evaluate vaccination coverage in red foxes and raccoon dogs. Further analysis demonstrated that significantly different levels of seroconversion were found for the two different kits. BioPro ELISA results showed lower seroconversion level than those of the Bio-Rad ELISA kit. These discrepancies are inconsistent with previous studies in which the seroconverion were found lower using Bio-Rad compared to BioPro kits due to the lower sensitivity of the first test [33,70]. Our results may be explained by the fact that a different cut-off value from the 0.5 conventional one’s was used for the Bio-Rad kit (0.125 instead of the 0.5 used in Wasniewski et al.). These results must be also nuanced by the fact that Latvia encountered specific events in the same period when using the Bio-Pro test in 2012, a year during which an epidemic sarcoptic mange occurred. Immunological reactivity due to sarcoptic mange could potentially have interfered with the rabies vaccination, leading to a lower response and a decrease in the level of rabies antibodies.

### Importance of considering age and species of sampled population to compare ORV monitoring results

A sharp decrease in the number of marked animals was observed in Estonia during the last four campaigns as soon as the ORV area was reduced to a buffer zone of 9 325 km^2^ (20 km along the Southern border and 30–50 km in eastern part of the country). This drop could be explained, inter alia, by an “edge effect” due to the small size of the vaccinated areas. The areas being small, the perimeter-to-surface ratio is higher and the probability of sampling an unvaccinated animal in bordering areas is higher than for a large ORV areas. Moreover, the proportion of raccoon dogs in the monitoring sample has increased every year, ultimately constituting more than ¾ of all animals tested. This example highlights the importance of considering the structure of the monitoring sample in the determinism of the overall and final bait uptake level. Thus, comparison of monitoring data between countries and their interpretation should be assessed by taking into account the species (raccoon dogs vs red foxes) and the age class (juvenile vs adult) of the sampled population.

### Phylogenetic analysis

The molecular epidemiology of RABV in the Baltic countries showed the presence of three types of RABV variants in the Baltic States, the North-East European group (NEE) (158/165 isolates), the Russian group (C) (5/165 isolates) and two vaccine-induced rabies cases. These results confirm that the terrain for rabies hosts infected with Baltic variants is broad [71], ranging from Eastern to the Central Europe. More precisely, the NEE group has been reported in Eastern part of Russia and from Finland to Romania [49] including the Baltic States [28,72,73], Slovakia, Poland and Ukraine [38,48,74], while C group has been reported from the European part of Russia [48] to different parts of Ukraine [74]. Although the C group is the most widely reported RABV variant in Russia [75] including regions of western Siberia, Kazakhstan and Tuva, four other variants have been previously described in Russia [48].

This study is the first to report the presence of C variant in North-East Europe with three cases in Lithuania reported between 2009 and 2013, one case in Estonia in 2011 and one case in Latvia in 2012. The occurrence of the C variant in Baltic States could be the result of a westward spread of rabies-infected hosts from Russia or from Belarus to the Baltic States. Animal-to-animal transmission of rabies virus or human-mediated transports of latently infected animals could explain the movement of rabies infected hosts across borders. There are numerous studies illustrating rabies virus transmission by human-mediated animal movements [76], wildlife-mediated movement of rabies [50] or movements of infected animals a cross frozen seas [77].

In Russia, six wild canid species (red fox, raccoon dog, artic fox, steppe fox, jackal and wolf) are vector of the disease. In Eastern Europe and in north-eastern Europe, most wildlife cases are reported in red foxes and raccoon dogs. The NEE variant is particularly associated with raccoon dogs in north-western Russia and north-eastern Europe, while C group were previously associated with the red fox and the steppe fox in Russia [48]. In this study, no phylogenetic distinction was reported between the red fox and raccoon dog isolates, whatever the variant analysed (C and NEE groups) and whatever the phylogenetic method used. Perfect identity observed between one isolate (red fox) in Estonia in 2006 and five strains (two raccoon dogs, one fox, one cattle and a dog) isolated in Latvia and in Lithuania between 2007 and 2009 suggests that the variant circulating in fox and raccoon dog populations have the same origin. Dogs may have served as an early reservoir for interspecies rabies virus transmission generating viral lineages that then spread to other species [78].

Due to the risk of residual pathogenicity of oral rabies vaccines related to the viral strain’s attenuation level, all rabies virus samples isolated in areas where attenuated rabies virus vaccines are used should be typed in order to distinguish between vaccine and field virus strains [2,19,22]. For the first time, we demonstrated that two field Baltic isolates (a marten from Lithuania in 2008 and a badger from Latvia in 2013), clustered with the group forming the rabies vaccines, SAG2, SAD B19 and SAD Bern. Clearly, the two vaccine-induced rabies cases were closely related to SAD B19 strains, although both cases were found in an area vaccinated with SAD Bern Lysvulpen baits. Previous study results indicated that the SAD Bern Lysvulpen vaccine shows higher similarity to the strains belonging to the SAD B19 vaccine [79]. Such findings led to a change in the viral strain description for the national marketing authorization dossier of this vaccine, http://www.uskvbl.cz/en/authorisation-a-approval/marketing-authorisation-of-vmps/list-of-vmps/authorised-by-national-and-mrdc-procedures.

This is also the first reporting of a vaccine-associated virus detected in badgers and in martens. To date, few vaccine-induced rabies cases have been documented in target species. Muller et al. [80] reported six vaccine-induced rabies cases in foxes caused by SAD B19 and SADP5/88 in vaccinated areas in Germany and Austria, respectively. In Slovenia, a young fox was also shown closely related to SAD B19 in 2012 [81]. The most likely explanation for these vaccine associated cases isolated in non target species is either residual pathogenicity of the virus vaccine despite vaccine attenuation or reversion to virulence. RNA viruses are known to have high mutation rates due to the lack of proofreading by RNA polymerases and could have occasionally reversed to more virulent viruses. The second hypotheses would be a transmission from a red fox or raccoon dog initially infected by a vaccine strain. Potential transmission of vaccine strain has indeed been recently questioned when finding vaccine strain in salivary gland of a naturally infected fox [81].

## Supporting Information

S1 Table Description of Baltic isolates and referenced sequences used in the phylogenetic analysis. ND: Not determined; NEE: North Eastern European phylogroup; WE: Western European phylogroup; CE: Central European phylogroup; D: Western Russian phylogroup; C: European part of the Russian phylogroup.
(DOCX)
