# Supplementary material for: Rabies in the Baltic States: Decoding a Process of Control and Elimination
Source: PLoS Negl Trop Dis. 2016 Feb 5;10(2):e0004432. doi: 10.1371/journal.pntd.0004432 (PMC4743931; doi:10.1371/journal.pntd.0004432)
Supplement: S1 Table — ND: Not determined; NEE: North Eastern European phylogroup; WE: Western European phylogroup; CE: Central European phylogroup; D: Western Russian phylogroup; C: European part of the Russian phylogroup. (DOCX) [file pntd.0004432.s001.docx]

Table 1

| N | Country | Sample  identification | Year  of isolation | Date  of  isolation | Species | District | Phylogroup | GenBank  accession  number |
| --- | --- | --- | --- | --- | --- | --- | --- | --- |
| 1 | Estonia | MT 720 | 2004 | ND | Raccoon dog | ND | NEE | JN109225 |
| 2 |  | 12767 MT 745 | 2004 | ND | Raccoon dog | ND | NEE | JN109217 |
| 3 |  | MT 740 | 2004 | ND | Raccoon dog | ND | NEE | JN109218 |
| 4 |  | MT 724 | 2004 | ND | Raccoon dog | ND | NEE | JN109226 |
| 5 |  | TR0600707, MT39 | 2006 | ND | Cat | ND | NEE | KR337548 |
| 6 |  | TR0603044, MT90 | 2006 | ND | Red fox | ND | NEE | JN109227 |
| 7 |  | TA07-05424, MT42 | 2007 | ND | Cattle | ND | NEE | JN09224 |
| 8 |  | BMT-4 | 2006 | ND | Raccoon dog | ND | NEE | JN109223 |
| 9 |  | TR06-01755 | 2006 | ND | Raccoon dog | ND | NEE | JN109229 |
| 10 |  | TR06-03634 | 2006 | ND | Red fox | ND | NEE | KR337546 |
| 11 |  | TR06-11366 | 2006 | ND | Raccoon dog | ND | NEE | JN109231 |
| 12 |  | TR06-11584 | 2006 | ND | Red fox | ND | NEE | KR337547 |
| 13 |  | TR06-12537 | 2006 | ND | Raccoon dog | ND | NEE | KR337549 |
| 14 |  | TR06-12855 | 2006 | ND | Red fox | ND | NEE | JN109232 |
| 15 |  | TR09-13593 | 2009 | ND | Red fox | ND | NEE | KR337550 |
| 16 |  | MT-3/TA11-00267 | 2011 | ND | Red fox | ND | C | KR337551 |
| 17 | Latvia | RV13 | 2004 | 04/11/2004 | Raccoon dog | Dobele | NEE | KR337511 |
| 18 |  | RV6 | 2004 | 19/07/2004 | Dog | Saldus | NEE | KR337512 |
| 19 |  | RV11 | 2004 | 09/08/2004 | Cow | Preili | NEE | KR337513 |
| 20 |  | RV12 | 2004 | 04/11/2004 | Raccoon dog | Kraslava | NEE | KR337514 |
| 21 |  | RV9 | 2004 | 22/07/2004 | Cow | Kraslava | NEE | KR337515 |
| 22 |  | RV3 | 2004 | 28/05/2004 | Raccoon dog | Kraslava | NEE | KR337516 |
| 23 |  | RV1 | 2004 | 24/03/2004 | Red fox | Riga | NEE | KR337517 |
| 24 |  | RV15 | 2008 | 22/01/2008 | Red fox | Liepājas | NEE | KR337518 |
| 25 |  | RV20 | 2008 | 17/02/2008 | Raccoon dog | Saldus | NEE | KR337519 |
| 26 |  | RV28 | 2008 | 02/12/2008 | Raccoon dog | Kuldīga | NEE | KR337509 |
| 27 |  | RV19 | 2008 | 22/08/2008 | Raccoon dog | Jēkabpils | NEE | KR337521 |
| 28 |  | RV7 | 2009 | 24/03/2009 | Raccoon dog | Saldus | NEE | KR337520 |
| 29 |  | RV12 | 2009 | 20/03/2009 | Raccoon dog | Talsi | NEE | KR337552 |
| 30 |  | RV13 | 2009 | 03/03/2008 | Raccoon dog | Talsi | NEE | KR337553 |
| 31 |  | RV28 | 2009 | 02/12/2008 | Raccoon dog | Kuldiga | NEE | KR337510 |
| 32 |  | 10753_A36 | 2009 | 27/05/2009 | Red fox | Rezeknes | NEE | JQ005109 |
| 33 |  | 30618-2011 | 2011 | 28/12/2011 | Horse | Kraslava | NEE | JQ894944 |
| 34 |  | 6909-2012 | 2012 | 06/02/2012 | Cattle | Dagda | C | JQ894943 |
| 35 |  | Latv_DR784 | 2013 | 10/04/2013 | Badger | Aloja | Rabies Vaccine Strain | KR337508 |
| 36 | Lithuania | 11768 | 2007 | 29/05/2007 | Dog | Sirvintai | NEE | KR337524 |
| 37 |  | 12537 | 2007 | 08/06/2007 | Cat | Moletai | NEE | KR337523 |
| 38 |  | 13382 | 2007 | 18/06/2007 | Red Fox |  | NEE | KR337525 |
| 39 |  | 25728 | 2007 | 23/11/2007 | Cat | Kaisiadoriai | NEE | KR337526 |
| 40 |  | 3085 | 2008 | 12/02/2008 | Red fox | Zarasai | NEE | KR337522 |
| 41 |  | 18200 | 2008 | 19/09/2008 | Raccoon dog | Varena | NEE | KR337527 |
| 42 |  | 24771 | 2008 | 18/11/2008 | Marten | Alytus | Rabies Vaccine Strain | KR337545 |
| 43 |  | 1064 | 2009 | 22/01/2009 | Raccoon dog | Ignalina | NEE | KR337544 |
| 44 |  | 5121 | 2009 | 23/03/2009 | Red fox | Vilnius | NEE | KR337528 |
| 45 |  | 5859 | 2009 | 07/04/2009 | Red fox | Varėna | NEE | KR337529 |
| 46 |  | 6052 | 2009 | 09/04/2009 | Raccoon dog | Ignalina | NEE | KR337530 |
| 47 |  | 6211 | 2009 | 15/04/2009 | Raccoon dog | Salcininkai | NEE | KR337531 |
| 48 |  | 6750 | 2009 | 27/04/2009 | Dog | Trakai | NEE | KR337532 |
| 49 |  | 13448 | 2009 | 29/09/2009 | Raccoon dog | Zarasai | NEE | KR337533 |
| 50 |  | 13658 | 2009 | 06/10/2009 | Raccoon dog | Vilnius | NEE | KR337534 |
| 51 |  | 13696 | 2009 | 09/10/2009 | Raccoon dog | Ignalina | NEE | KR337535 |
| 52 |  | 14238 | 2009 | 19/10/2009 | Cattle | Kretinga | NEE | KR337536 |
| 53 |  | 14541 | 2009 | 26/10/2009 | Raccoon dog |  | NEE | KR337537 |
| 54 |  | 16571 | 2009 | 07/12/2009 | Red fox |  | C | KR337538 |
| 55 |  | 17143 | 2009 | 18/12/2009 | Raccoon dog | Klaipeda | NEE | KR337539 |
| 56 |  | 4740 | 2010 | 14/04/2010 | Raccoon dog | Varena | C | KR337540 |
| 57 |  | 3747 | 2012 | 15/02/2012 | Cat | Salcininkai | NEE | KR337541 |
| 58 |  | 16392 | 2012 | 24/03/2012 | Cattle | Ignalina | NEE | KR337542 |
| 59 |  | 864 | 2013 | 15/01/2013 | Dog | Varėna | C | KR337543 |
| Poland | |  |  |  |  |  | NEE | JN190391 |
|  |  |  |  |  |  |  | NEE | AF033875 |
|  |  |  |  |  |  |  | NEE | AF033879 |
|  |  | 96250 |  |  |  |  | CE | AF033887 |
|  |  |  |  |  | Fox |  | CE | JN190359 |
|  |  | 96176 |  |  |  |  | EE | AF0333883 |
| Russia | |  |  |  | Cat |  | C | AY352503 |
|  |  |  |  |  | Fox |  | C | AY352510 |
|  |  |  |  |  | Fox |  | D | AY352511 |
|  |  |  |  |  | Fox |  | D | AY352506 |
|  |  |  |  |  | Dog |  | D | AY352508 |
|  |  |  |  |  | Dog |  | D | AY352476 |
| Ukraine | | RV09-004 | 2009 |  | Cat | Dnipropetrovsk | NEE | JN656493 |
|  |  | RV08-007 | 2008 |  | Stray dog | Sumy | C | JN656487 |
| Romania | | RO-RV-1281-06-IL | 2006 |  | Domestic dog |  | NEE | GU086632 |
| Slovakia | | 94250 | 1994 |  | Cat |  | NEE | U43007 |
| Bulgaria | |  |  |  | Fox |  | EE | DQ300294 |
| Hungary | | 9386 |  |  | Fox |  | EE | U43000 |
| Croatia | |  |  |  | Wolf |  | WE | GU134624 |
| Bosnia | |  |  |  | Fox |  | WE | U42706 |
| TCE | | 9465 |  |  |  |  | CE | U43010 |
| CVS | |  |  |  |  |  |  | D42112 |
| ptzn | |  |  |  |  |  |  | HQ829841 |
| Artic isolates : | | | | | | | | |
| Greenland | | 8684 |  |  | Artic fox |  |  | U22654 |
| Belarus | | RV441 |  |  | Fox |  |  | DQ010126 |
| Russia | | RV1338 |  |  | Artic fox |  |  | DQ10131 |
| Artic-like isolates : | | | | | | | | |
| Russia | | RV303 |  |  | Raccoon dog |  |  | AY352505 |
| Russia | | 857 |  |  | Raccoon dog |  |  | AY352458 |
| Russia | | 248 |  |  | Fox |  |  | AY352460 |
| Rabies vaccines : | | | | | | | | |
| SAD Bern | |  |  |  |  |  |  | EF206708 |
| SAD B19 | |  |  |  |  |  |  | EF206709 |
| SAG2 | |  |  |  |  |  |  | E206719 |
